# Bone marrow brews central nervous system inflammation and autoimmunity

**DOI:** 10.1002/ctm2.1125

**Published:** 2022-11-22

**Authors:** Mingming Liu, Qiang Liu

**Affiliations:** ^1^ Department of Neurology Tianjin Neurological Institute Tianjin Medical University General Hospital Tianjin China

**Keywords:** autoimmunity, bone marrow, central nervous system, inflammation

## 1 BONE MARROW AND MULTIPLE SCLEROSIS (MS)

Multiple sclerosis (MS) is a T‐cell‐mediated chronic inflammatory disease of the central nervous system (CNS) that manifests in young adults as a predominant cause of severe neurological disability, which imposes a heavy economic and psychological burden on the patient's family.^1^ Currently, there are many shortcomings in the efficacy of drugs to control the progression of the disease. These unmet clinical needs stem from incompletely understood immune mechanisms of MS. Past research on autoreactive T cells in MS patients has been limited to blood and cerebrospinal fluid.^2^ Of note, the location and factors that determine the initiation and evolution of autoreactive T cells, and the impact of these factors on the progression of MS disease, are still poorly understood. Bone marrow harbours haematopoietic stem and progenitor cells (HSPCs) that give rise to all immune cell types.^3^ Recent evidence has demonstrated the skull and vertebral bone marrow as a key reservoir of myeloid cells and lymphocytes for the meninges and CNS parenchyma.^4^, ^5^ These findings are partially supported by a previous study showing skull bone marrow and the brain surface are connected by direct vascular channels that enable myeloid cell migration into brain.^6^ Emerging evidence indicates that infectious or inflammatory stimuli induces adaptation of bone marrow HSPCs, leading to an increase in the output of myeloid cells by activation of lineage‐specific transcription factors.^7^ These observations suggest a potential link between the activation of bone marrow HSPCs and the co‐operation between the innate and adaptive immunity, raising several interesting questions particularly including whether this occurs in patients with chronic inflammatory diseases and its impact on disease outcome.^8^

## 2 ABERRANT BONE MARROW MYELOPOIESIS AND INCREASED T‐CELL CLONAL EXPANSION IN PATIENTS WITH MS

The aetiology of MS is focused on myelin‐reactive T cells that infiltrate the CNS to cause demyelinating lesions.^9^ One recently study coupled single cell sequencing and flow cytometry analysis of bone marrow HSPCs and their downstream cellular lineages in MS patients.^8^ The authors found that bone marrow HSPCs were predominantly skewed towards myeloid lineage concomitant with an increase of transcription factors in myeloid lineage but not lymphoid lineage. This finding is unexpected because MS is thought to be a T‐cell‐mediated autoimmune CNS inflammatory disease and the alterations in bone marrow myeloid cells have not been comprehensively depicted. Notably, the augmented myelopoiesis in bone marrow of MS patients was accompanied by enhanced clonal expansion of T cells. These and other observations led the authors to conclude that bone marrow HSPCs can sense and adapt to systemic immune activation in MS patients, a process that drives CNS inflammation and autoimmunity.

## 3 RESET BONE MARROW IMMUNITY TO RESTORE IMMUNE HOMEOSTASIS IN MS

**FIGURE 1:**
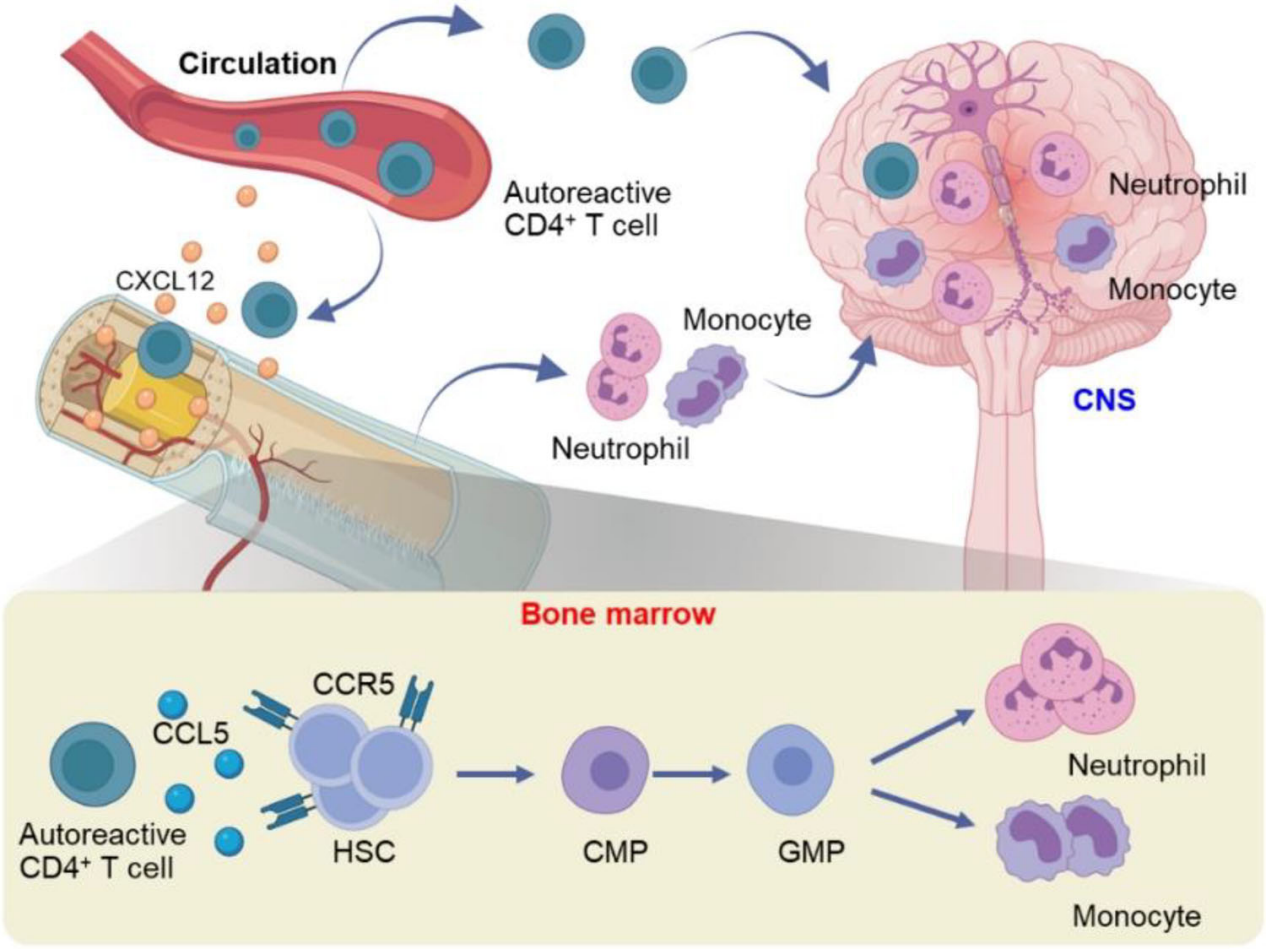
Autoreactive T cells migrate into bone marrow to instruct haematopoiesis that propagates neuroinflammation. In multiple sclerosis (MS), peripheral autoreactive CD4^+^ T cells expressing CXCR4 are guided by CXCL12 to migrate into bone marrow niche. In the bone marrow, autoreactive T cells rewire HSPCs towards myeloid cellular lineages via CCL5‐CCR5 axis, leading to augmented production of neutrophils and monocytes. Subsequently, newly generated neutrophils and monocytes leave bone marrow and enter CNS to accelerate inflammatory CNS injury during early disease development. Blocking of CCL5‐CCR5 axis ablates detrimental bone marrow myelopoiesis, and thereby curb inflammatory CNS injury. HSC: haematopoietic stem cell, CMP: common myeloid progenitor, GMP: granulocyte‐monocyte progenitor, CXCL12: C‐X‐C Motif Chemokine Ligand 12, CCL5: C‐C motif chemokine ligand 5, CCR5: C‐C Chemokine Receptor 5

To determine the fate of HSPCs and their downstream cellular lineages, the authors mirrored these observations in experimental autoimmune encephalomyelitis, a mouse model of MS. Lineage tracing revealed that increased bone marrow myelopoiesis led to augmented output of monocytes and neutrophils that subsequently invaded the CNS. Of interest, they showed that myelin‐reactive T cells migrated into the bone marrow compartment in a CXCL12‐CXCR4 dependent manner. Subsequently, the myelin‐reactive T cells highly expressed CCL5, which resulted in bone marrow myelopoiesis in a CCL5‐CCR5 axis dependent manner (Figure 1). The aberrant bone marrow myelopoiesis was ablated in bone marrow chimeric mice reconstituted with CCR5^−/‐^ HSCs or wild type mice receiving a CCR5 inhibitor, suggesting a detrimental role of CCL5‐CCR5 axis in myelopoiesis that drives CNS inflammation and demyelination.^8^

## 4 FUTURE OUTLOOK

These findings extend the previous understanding of autoreactive T cells within lymph organs and CNS into bone marrow in MS, implicating bone marrow as a treatment target to reset and correct the aberrant immune response leading to CNS autoimmunity in MS. This notion is supported by results from clinical trials, in which autologous haematopoietic stem cell transplantation, i.e. bone marrow transplantation, can arrest neurological deterioration and prolong medication‐free interval in patients with aggressive MS.^10^ Given that immune suppressant drugs to treat MS often have side effects of suppressing myelopoiesis, it seems plausible to speculate that the reduced myelopoiesis may actually contribute to the benefit of these medications. Future investigations are required to identify possible therapies targeting bone marrow to restore immune homeostasis, and thus to control CNS autoimmune neuroinflammation.

## CONFLICT OF INTEREST

The authors declare no competing interests exist.
